# Dynamic immune and exosome transcriptomic responses in patients undergoing psychostimulant methamphetamine withdrawal

**DOI:** 10.3389/fncel.2022.961131

**Published:** 2022-09-27

**Authors:** Hongjin Wu, Zunyue Zhang, Yuru Ma, Fengrong Chen, Pu Xiong, Zhenrong Xie, Guo Ding, Juehua Yu, Kunhua Wang

**Affiliations:** ^1^School of Medicine, Yunnan University, Yunnan, China; ^2^NHC Key Laboratory of Drug Addiction Medicine, Kunming Medical University, Kunming, China; ^3^International Research Center for Regenerative Medicine, BOAO International Hospital, Qionghai, China; ^4^High School Attached to Shanghai Normal University, Shanghai, China

**Keywords:** methamphetamine, exosome, transcriptome, regulatory network, immune response

## Abstract

Methamphetamine (METH) addiction and withdrawal cause serious harm to both the immune system and nervous system. However, the pathogenesis remains largely unknown. Herein, we investigated the peripheral cytokines and exosomal transcriptome regulatory networks in the patients with METH use disorders (MUDs) undergoing withdrawal. Twenty-seven cytokines were simultaneously assessed in 51 subjects, including 22 at the acute withdrawal (AW) stage and 29 at the protracted withdrawal (PW) stage, and 31 age and gender-matched healthy controls (HCs). Compared to the HCs, significantly decreased levels of interleukin (IL)-1β, IL-9, IL-15, Basic FGF, and MIP1a, increased levels of IL-1rα, IL-6, Eotaxin IP-10, VEGF, and RANTES were identified in AW. These disturbances were mostly or partly restored to the baseline in PW. However, the cytokines IL-6, IL-7, and IL-12p70 were consistently increased even after one year of withdrawal. Besides, a significant decrease in CD3^+^T and CD4^+^T cell numbers was observed in AW, and the diminishment was restored to baseline in PW. Comparatively, there were no statistically significant changes in CD8^+^T, NK, and B cells. Furthermore, the exosomal mRNAs and long non-coding RNAs (lncRNA) were profiled, and the lncRNA-miRNA-mRNA networks were constructed and associated with METH AW and PW stages. Notably, the chemokine signaling was remarkably upregulated during AW. By contrast, the differentially expressed mRNAs/lincRNAs were significantly enriched in neurodegeneration-related diseases. Taken together, a group of METH withdrawal-related cytokines and exosomal mRNA/lncRNA regulatory networks were obtained, which provides a useful experimental and theoretical basis for further understanding of the pathogenesis of the withdrawal symptoms in MUDs.

## Introduction

Methamphetamine (METH) is one of the most common and highly addictive psychostimulants that cause severe damage to a wide spectrum of organ systems (Darke et al., 2008; Glasner-Edwards et al., 2010). In addition to those METH induced neuropsychiatric and neurodegenerative symptoms such as depression, anxiety, and/or cognitive impairments (Hall et al., 1996; McKetin et al., 2008), methamphetamine use disorders (MUDs) could also give rise to considerable problems associated with immunoglobins and cytokine/chemokine signaling pathways, which may contribute to spread of devastating infectious diseases (Potula et al., 2010; Harms et al., 2012) and thereafter influence the recovery from neurodegeneration defects (Loftis and Huckans, 2013). To date, despite the astounding efforts into the knowledge base of addiction medicine, the underlying molecular mechanism and functional interplay between immune response and neurodegeneration remain largely lacking (Zhang et al., 2021; Zhou et al., 2021).

The development of omics-based high-throughput technologies nowadays allows for a simultaneous determination of a large number of potential biomarkers and their correlations to the disease progressions (Hoshino et al., 2020). For example, the cytokine multiplex assay allows researchers to evaluate the concentrations of multiple cytokines of interest in a limited sample volume (Lynch et al., 2014). Likewise, exosomes are tiny endosomal-derived membrane micro-vesicles that contain a large amount of regulatory information and may participate in a series of basic biological processes (Beatriz et al., 2021; Qiu et al., 2021). Compared to those diluted nucleotide pieces in the peripheral blood, the exosomal content is much more stable. Therefore, the value of analyzing exosome-encapsulated DNA/RNA has been recognized (Cheng et al., 2015; Ramirez et al., 2018), and the exosomes in liquid biopsy are commonly utilized to develop diagnostic tools in clinical applications (Grieco et al., 2021; Masoumipour et al., 2021; Zani-Ruttenstock et al., 2021). Among these, studies have shown that exosomes could be used for predicting Alzheimer’s disease (AD) and evaluating the severity of intellectual disability in patients with Parkinson’s disease and dementia (Cheng et al., 2015; Stuendl et al., 2016). As for MUDs, the disease management would take advantage of having adequate peripheral biomarkers and gain greater insight into immune response in patients undergoing METH withdrawal.

We had already identified those differentially expressed long non-coding RNAs (lncRNAs) and mRNAs in the plasma exosomes of heroin addicts, which are specifically associated with heroin withdrawal stages and the irregularity of both adaptive and innate immunity (Zhang et al., 2021). The present study was designed to investigate the pattern of METH-withdrawal altered immune responses, and to unveil the function of circulating exosome capsulated molecules in patients with MUDs. Our present approach aims to uncover the mechanism associated with the peripheral exosomal biomarkers and transcriptome regulatory network in developing withdrawal symptoms.

## Materials and Methods

### Study participants and ethics statement

In order to analyze the dynamics of the cytokines in MUDs, we recruited 82 male participants in the Kunming Drug Rehabilitation Center between January 2018 and October 2019, including 51 MUDs patients and 31 healthy control subjects. For exosomal RNA sequencing analysis, 20 MUDs consisting of 10 subjects each at 7 to 14-day and 1-year stages of METH withdrawal, and 10 healthy individuals were recruited and previously described in Chen et al. (2021) and Yu et al. (2021). All participants provided written informed consent before enrollment. The recruitment procedures and protocols were approved by the Research Ethics Committee of the First Affiliated Hospital of Kunming Medical University (2018-L-42). The drug use history of patients with MUDs was obtained by self-report and verified with caregivers. Samples were collected as previously described (Zhang et al., 2021).

The inclusion criteria were as follows: (1) patients were diagnosed with methamphetamine dependence, (2) patients were between the ages of 20 and 55. The exclusion criteria were defined as: (1) patients with medical or neurological disease or trauma affecting their central nervous system, (2) patients who have been reported to have a history of HIV or other infectious diseases, (3) patients who had severe endocrine, cardiovascular, or a history of loss of consciousness for more than 30 min.

### Determination of peripheral cytokine levels in human plasma

Fresh whole blood samples were collected from the study participants using a 10 ml EDTA-2Na vacuum tubes. The blood samples were centrifuged at 1,500 *g* for 15 min, and the plasma was transferred into a new tube. The plasma was centrifuged at 20,000 *g* at 4°C for 15 min to remove platelets. Purified plasma was harvested, aliquoted, and stored at −80°C until assay.

The Luminex Human Cytokine Assay was performed using the Luminex Human Magnetic Assay Kit (R&D Systems, MN, USA) according to the manufacturer’s instructions. Standard curves were generated by Bio-plex Manager software to determine unknown sample concentrations. The detection kit includes 27 cytokines, including bFGF, IL-1β, IL-2, IL-4, IL-5, IL-6, IL-7, IL-8, IL-9, IL-10, IL-12p70, IL-13, IL-15, IL-17A, IP-10, IFN-γ, MCP-1, MIP-1α, MIP-1β, PDGF-BB, RANTES, TNF-α, and VEGF.

### Immune cell subsets

Fresh whole blood samples were analyzed with flow cytometry. For flow cytometry, we followed the protocol using 50 μl of whole blood and added 5 μl of monoclonal antibody, then incubated for 15 min at room temperature in the dark. Added 800 μl BD 1× lysis solution to each sample and incubated for 10 min at room temperature in the dark. The sample was centrifuged at 500 *g* for 5 min, and the pellet was collected and resuspended with 300 μl of FACS Flow, and analyzed with BD FACS Canto II cytometer.

The antibody used in this study was BD Multitest^TM^ 6-color TBNK Reagent (Cat# 644611), including CD3-FITC/CD4-PE-Cy7/CD8-APC-Cy7/CD16&CD56-PE/CD19-APC/CD45-PerCP-Cy5.5 (BD, United States). The absolute cell counts were calculated by multiplying the percentage of immune cell subsets by the concentration of total lymphocytes present in the peripheral blood.

### Exosomal RNA sequencing and identification of differentially expressed lncRNA and mRNA

The exosomes from peripheral blood were isolated as previously described (Chen et al., 2021). The Exosupur^®^ columns (Echobiotech, China) were used to purify 2 ml of 0.8 μm-filtered plasma samples followed by elution of exosomes with PBS. The eluted fractions were concentrated to 200 μl using Amicon^®^ Ultra spin filters with a molecular weight cut-off of 100 kDa (Merck, Germany). Total RNAs were then extracted from the purified exosomes using the miRNeasy^®^ Mini kit according to the manufacturer’s protocol, then qualified with Agilent Bioanalyzer 2100 (Agilent Technologies, Inc., CA, USA).

For RNA sequencing library construction, 1.5 μg RNA per sample was used as input material. The rRNA was removed using the Ribo-Zero rRNA Removal Kit (Epicentre, Madison, WI, USA). The sequencing libraries were generated using NEBNext Ultra^TM^ Directional RNA Library Prep Kit for Illumina (NEB, USA) following the manufacturer’s instructions. Raw reads of RNA-seq data were filtered using fast QC, then aligned to the GRCh38 human genome assembly using HISAT2 (Pertea et al., 2016). Annotations of mRNA and lncRNA in the human genome were retrieved from the GENCODE (v.25). The mRNAs and lncRNAs were quantified and analyzed using DESeq2 R package (Love et al., 2014) and StringTie 1.3.1 (Pertea et al., 2016), respectively.

### Functional and pathway enrichment

Gene Ontology (GO) and KEGG pathways enrichment analyses were performed in DAVID website^1^ that are significantly enriched compared to the entire human genome background (Huang da et al., 2009; Sherman et al., 2022).

### Prediction of miRNA/lncRNA targets and lncRNA-miRNA-mRNA regulatory network construction

The targets of DE miRNAs were analyzed by miRTarBase, a database containing targets validated experimentally (Huang et al., 2020). To summarize the overlap between predicted mRNA targets and DE mRNAs, Venn diagrams were constructed by FunRich 3.1 software. LncRNAs that could regulate miRNA expression were predicted by DIANA-LncBase v3^2^ (Karagkouni et al., 2020). The lncRNA-miRNA-mRNA regulatory network was constructed according to the prediction results of lncRNA targets and miRNA targets, and the interaction between lncRNA, miRNA, and mRNA was displayed. Cytoscape software was used to plot the lncRNA-miRNA-mRNA regulatory network.

### Data availability and data analysis

All sequencing data have been deposited in the Gene Expression Omnibus (GEO) database under the accession number GSE172306. *Kruskal–Wallis* test or analysis of variance (ANOVA) test followed by a *post-hoc* test (Bonferroni’s *t*-test) was used to test for differences in continuous variables. Comparison between groups was performed using a student’s *t*-test or a Chi-square analysis as appropriate. The Pearson correlation was used for correlation analyses.

## Results

### Clinical characteristics

To characterize the immune responses in patients undergoing METH withdrawal, we recruited 51 male patients with MUDs, of which 22 and 29 subjects were approximately 7 to 14 days (acute withdrawal stage, AW) and 1 year (protracted withdrawal stage, PW) after the initiation of the abstinence from METH, respectively, as well as 31 age-matched healthy male volunteers (healthy controls, HC). The main clinical characteristics of the study participants were summarized in Table 1. There were no significant differences for any variables, including age, substance-use history, and education level, between the two groups of MUDs and HCs.

**Table 1:** Demographic and clinical characteristics of methamphetamine addicts (*n* = 51) and health controls (*n* = 31).

	**Healthy control**	**7–14 day**	**1 year**	*p*
	*n* = 31	*n* = 22	*n* = 29	
**Age**	35.61 ± 9.63	33.32 ± 6.64	37.87 ± 6.84	0.058
∣rule
**Drug history (year)**	NA	8.27 ± 4.91	12.31 ± 6.01	**0.0329**
**Education**				
Illiteracy-primary school	14 (45.16%)	16 (72.72%)	10 (34.48%)
Junior high school	9 (29.03%)	4 (18.18%)	15 (51.72%)
High school	6 (19.35%)	2 (9.09%)	3 (10.34%)
College	2 (6.45%)	0	1 (3.45%)
**Drug type**				
Methamphetamine	NA	11 (50.00%)	9 (31.03%)	0.1262
Mixed absorption	NA	11 (50.00%)	20 (68.97%)
**Route**				
injection	NA	0	2 (6.90%)	0.3176
snorting	NA	21 (95.45%)	27 (93.10%)
others	NA	1 (4.55%)	0

Healthy controls (HCs): healthy individuals recruited for this study. 7–14 day (AW, acute withdrawal): Methamphetamine addicts who have been abstinent for 7–14 days. 1 year (PW, protracted withdrawal): Methamphetamine addicts who have been abstinent for 1 year. Age: the age of the individuals. Drug history (year): history of the drug addicts. Education: education levels of the drug addicts. Drug type: types of drug addicts. Route: drug delivery use by drug addicts. Values in bold indicate: 7–14 day (AW, acute withdrawal): Methamphetamine addicts who have been abstinent for 7–14 days.

### Alterations in plasma cytokine levels and immune cell subsets in patients undergoing METH withdrawal

The alterations of 27 cytokines in the plasma associated with METH withdrawal were determined using Luminex Human Cytokine 27-plex assay (see “Materials and methods” Section). IFN-γ and GM-CSF were excluded from analysis in the dataset because these two fell below the lower level of quantitation in >20% of samples. The concentrations of IL-8, IL-17A, G-CSF, MIP-1β, and MCP-1 in the plasma of MUD patients at both AW and PW were not significantly different from those in the HCs. In the AW subjects, compared with those in the HCs, significantly lower levels of IL-1β (*p* = 4.88E-11), IL-9 (*p* = 2.16E-10), IL-15 (*p* = 2.26E-03), Basic FGF (*p* = 1.90E-12), and MIP-1α (*p* = 1.01E-2) were observed (Table 2). Synchronously, there were remarkable increases in the levels of IL-1rα (*p* = 2.09E-02), IL-6 (*p* = 4.82E-04), IL-10 (*p* = 1.65E-02), IL-12P70 (*p* = 2.38E-03), Eotaxin (*p* = 9.07E-07), IP-10 (*p* = 1.07E-02), VEGF (*p* = 7.78E-03) and TANTES (*p* = 2.83E-04) in the AW subjects than in the HCs (Table 2). Compared with those in the HCs, significantly higher levels of IL-2 (*p* = 5.73E-03), IL-4 (*p* = 3.05E-02), IL-6 (*p* = 1.89E-03), IL-7 (*p* = 8.27E-04), IL-10 (*p* = 8.27E-04), IL-12P70 (*p* = 2.29E-02), IP-10 (*p* = 2.83E-02), TNFα (*p* = 6.50E-05), and VEGF (*p* = 1.25E-02; Table 2) were found in the PW subjects.

**Table 2:** Alterations in plasma cytokine levels in patients undergoing METH withdrawal.

	**Healthy Control**	**AW**	**PW**	**HCs vs. AW**			**HCs vs. PW**		
				**fold change**	**p.value**	**p.adj.**	**fold change**	**p.value**	**p.adj.**
IL-1rα	362.71 ± 159.70	704.28 ± 407.63	391.58 ± 163.54	0.34	5.68E-03	**2.09E-02**	0.95	6.29E-01	6.29E-01
IL-1β	7.61 ± 2.05	1.66 ± 2.53	8.16 ± 2.60	4.58	3.25E-11	**4.88E-11**	0.93	3.66E-01	3.66E-01
IL-2	7.55 ± 2.90	5.83 ± 3.15	10.54 ± 4.30	1.29	4.94E-02	7.41E-02	0.72	2.87E-03	**5.73E-03**
IL-4	3.88 ± 1.40	4.74 ± 4.34	4.73 ± 1.37	0.82	3.81E-01	4.57E-01	0.82	2.03E-02	**3.05E-02**
IL-6	1.59 ± 0.99	10.69 ± 8.75	6.26 ± 6.50	0.15	8.04E-05	**4.82E-04**	0.25	6.30E-04	**1.89E-03**
IL-7	79.08 ± 32.52	148.56 ± 63.29	136.90 ± 71.18	0.53	5.48E-05	**3.29E-04**	0.58	2.76E-04	**8.27E-04**
IL-8	23.33 ± 12.55	35.22 ± 23.86	23.90 ± 10.61	0.66	4.10E-02	9.49E-02	0.98	8.50E-01	9.06E-01
IL-9	989.96 ± 144.05	361.76 ± 271.80	1,032.11 ± 166.57	2.74	7.21E-11	**2.16E-10**	0.96	3.01E-01	3.01E-01
IL-10	5.91 ± 4.11	21.04 ± 23.24	12.93 ± 6.43	0.28	1.37E-02	**1.65E-02**	0.46	8.45E-06	**2.53E-05**
IL-12P70	2.57 ± 0.78	5.41 ± 3.14	7.83 ± 9.83	0.48	3.96E-04	**2.38E-03**	0.33	7.64E-03	**2.29E-02**
IL-15	302.97 ± 205.06	149.52 ± 130.94	326.32 ± 139.49	2.03	1.88E-03	**2.26E-03**	0.93	6.12E-01	6.12E-01
IL-17A	15.10 ± 7.21	14.65 ± 6.41	20.92 ± 13.33	1.03	8.12E-01	8.12E-01	0.72	4.34E-02	6.52E-02
Basic FGF	312.25 ± 100.21	95.68 ± 68.56	307.4 ± 90.32	3.26	1.26E-12	**1.90E-12**	1.02	8.44E-01	8.44E-01
G-CSF	283.81 ± 72.67	428.69 ± 316.89	301.72 ± 66.56	0.66	4.66E-02	6.99E-02	0.94	3.23E-01	3.23E-01
Eotaxin	149.88 ± 79.03	609.80 ± 301.75	143.19 ± 53.98	0.25	4.03E-07	**9.07E-07**	1.05	7.02E-01	7.02E-01
MIP-1α	4.68 ± 3.01	2.84 ± 1.70	5.97 ± 2.51	1.65	6.74E-03	**1.01E-02**	0.78	7.54E-02	7.54E-02
MIP-1β	286.61 ± 41.94	258.09 ± 111.30	292.92 ± 42.70	1.11	2.63E-01	3.15E-01	0.98	5.66E-01	5.66E-01
IP-10	2,614.77 ± 679.55	7,057.41 ± 6,339.60	4,318.66 ± 3,460.52	0.37	3.57E-03	**1.07E-02**	0.61	1.42E-02	**2.83E-02**
TNFα	73.93 ± 13.82	105.35 ± 41.46	100.50 ± 25.40	0.7	2.20E-03	**4.41E-03**	0.74	1.08E-05	**6.50E-05**
VEGF	463.08 ± 199.01	1,773.49 ± 1534.50	610.96 ± 217.65	0.26	2.87E-03	**7.78E-03**	0.76	1.04E-02	**1.25E-02**
RANTES	21,249.9 ± 42,921	79,128.9 ± 67,494.2	10,060.27 ± 36,543.35	0.1	9.88E-05	**2.83E-04**	0.83	8.50E-01	8.50E-01
MCP-1	68.64 ± 30.61	94.39 ± 77.73	0.161.51 ± 22.14	0.73	1.52E-01	1.83E-01	1.12	3.03E-01	3.03E-01

Healthy control (HCs): healthy individuals recruited for this study. AW (7–14 day): Methamphetamine addicts who have been abstinent for 7–14 days. PW (1 year): Methamphetamine addicts who have been abstinent for 1 year. HCs vs. AW: alterations of the plasma cytokine levels in AW stage. HCs vs. PW: alterations of the plasma cytokine levels in PW stage. Fold change: fold change of the alterations. p.value: p-value of the alterations. p.adj.: adjusted p-value of the alterations. Values in bold indicate: 7–14 day (AW, acute withdrawal): Methamphetamine addicts who have been abstinent for 7–14 days.

After a period of withdrawal to PW, the abnormal cytokines (e.g., IL-1rα, IL-1β, IL-9, IL-15, Basic FGF, Eotaxin, MIP-1α, and TANTES) could be restored to the normal range, however, several cytokines, IL-6, IL-7, IL-10, IL-12P70, IP-10, TNFα, and VEGF could only be restored partially after one year of withdrawal. Besides, it was worth noting that the levels of IL-2 and IL-4 were normal range in AW, but dysregulated in the PW stage in patients with MUDs.

**Figure 1:**
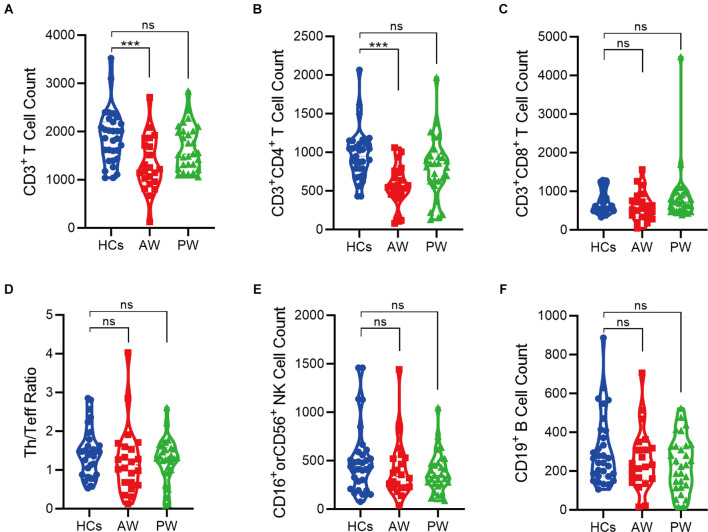
Alterations of peripheral immune cell subsets in patients undergoing methamphetamine (METH) withdrawal. **(A–F)** Total T cell count (CD3^+^) **(A)**, Th cell count (CD3^+^CD4^+^) **(B)**, Teff cell count (CD3^+^CD8^+^) **(C)**, Th/Teff ratio (CD3^+^CD4^+^/ CD3^+^CD8^+^) **(D)**, NK cell count (CD16^+^ or CD56^+^) **(E)**, and B cell count (CD19^+^) **(F)** in the MUDs patients during withdrawal. HCs, healthy controls; AW, acute withdrawal; PW, protracted withdrawal. ***p < 0.001; ns, no significant difference.

Furthermore, the immune cell subsets in the patients of MUDs during both AW and PW stages were analyzed (Figure 1 and Supplementary Figure S1). The absolute counts of CD3^+^T (*p* = 0.0004) and CD3^+^CD4^+^T (*p* = 0.0001) cells from the AW group were significantly lower than those of the HCs. A similar tendency was also observed for CD3^+^CD8^+^T cells, although there was no statistical difference. Considering that CD3^+^T cells are composed of CD3^+^CD4^+^T and CD3^+^CD8^+^T, these results suggest the tendency of CD3^+^T is dependent on CD3^+^CD4^+^T. In addition, the absolute counts of CD3^+^CD4^+^T were not fully recovered even one year after initiation of METH abstinence.

### Differential expression of exosomal mRNAs and lncRNAs at acute METH withdrawal stage

**Figure 2:**
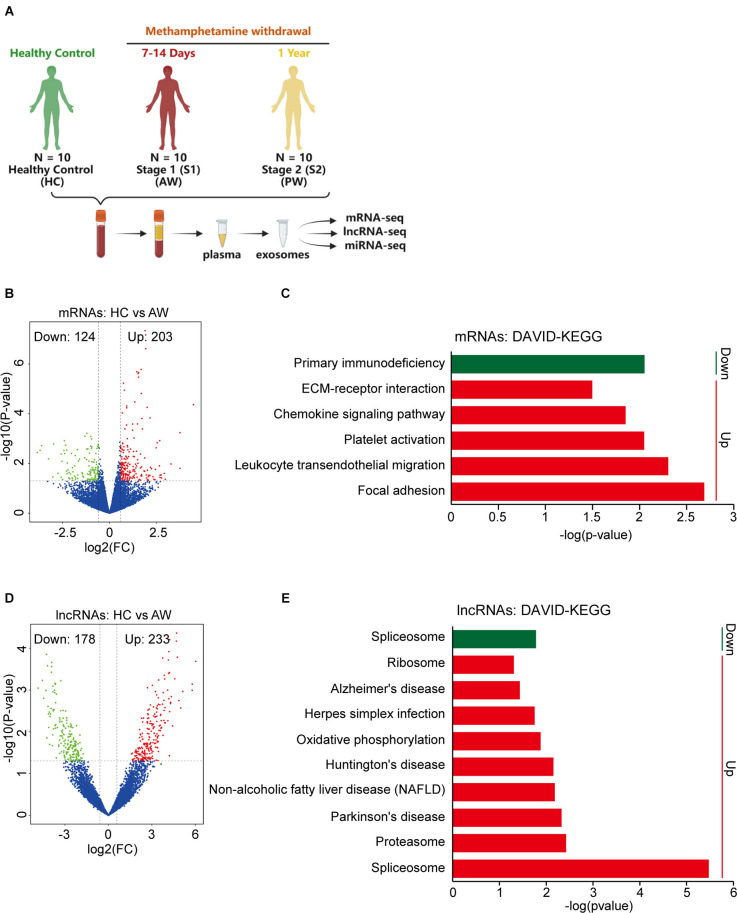
Differential expression of exosomal mRNAs and lncRNAs at acute METH withdrawal (AW) stage. **(A)** Sample collection and high-throughput sequencing processing schedule for exosomal transcriptome alterations in MUD patients. **(B)** Volcano plot showed the different expression (DE) mRNAs at the AW stage. **(C)** DAVID-KEGG analysis of DE mRNAs at AW stage. **(D)** Volcano plot showed the DE lncRNAs at AW stage. **(E)** DAVID-KEGG analysis of DE lncRNAs at AW stage. Green represents downregulated genes; Red represents upregulated genes. mRNA, messenger RNA.

To investigate the plasma exosomal mRNA/lncRNA expression changes associated with METH withdrawal, we additionally recruited 20 MUDs currently undergoing withdrawal (10 AW subjects and 10 PW subjects), and then compared their transcriptome with 10 HCs (Figure 2A). The clinical information of these study participants as well as the isolation and validation of exosomes from plasma samples were previously described (Yu et al., 2021). The population’s basic information variables, such as age, BMI, substance-use history, and education level were well balanced between the two groups of MUDs and the HCs (Supplementary Table S1).

The expression profiles of exosomal mRNA/lncRNA in the peripheral blood were determined by a high-throughput sequencing technique. We first compared the HC group vs. the AW group (S1), to identify the mRNAs/lncRNAs that were specifically responsible for the acute METH withdrawal stage. At the mRNA level, a total of 327 differentially expressed (DE) mRNAs, of which 124 downregulated and 203 upregulated were associated with the AW stage (Figure 2B). DAVID GO term analysis of these overlapped DE mRNAs showed that the platelets were persistently impaired in the AW stage. Genes involved in the biological process terms including* platelet degranulation* (15 genes, *p* < 0.0001), *platelet activation* (five genes, *p* = 0.0008), and *platelet aggregation* (six genes, *p* = 0.007) were significantly upregulated (Supplementary Table S2). Furthermore, KEGG pathway analysis indicated that these DE mRNAs were significantly enriched in the *Chemokine signaling pathway* (Increased, *p* = 0.014), *Leukocyte transendothelial migration* (Increased, *p* = 0.005), *Platelet activation* (Increased, *p* = 0.009), and *Primary immunodeficiency* (Decreased, *p* = 0.009; Figure 2C and Supplementary Table S2).

Next, we compared the lncRNA expression profiles between the HC group vs. the AW group. Using the threshold (|log2 (fold change)| > 0.5, *p* < 0.05), a total of 411 DE lncRNAs (233 downregulated and 178 upregulated lncRNAs) were identified to associate with METH AW (Figure 2D). GO and KEGG analyses of these DE lncRNAs suggested that the neurodegeneration diseases-related lncRNAs were significantly upregulated in the AW group, including Parkinson’s disease (*p* = 0.005), Huntington’s disease (*p* = 0.007) and Alzheimer’s disease (*p* = 0.04; Figure 2E and Supplementary Table S3). Overall, these results suggest that both the nervous system and the immune systems were significantly damaged during METH AW.

### Differential expression of exosomal mRNAs and lncRNAs during the protracted METH withdrawal

**Figure 3:**
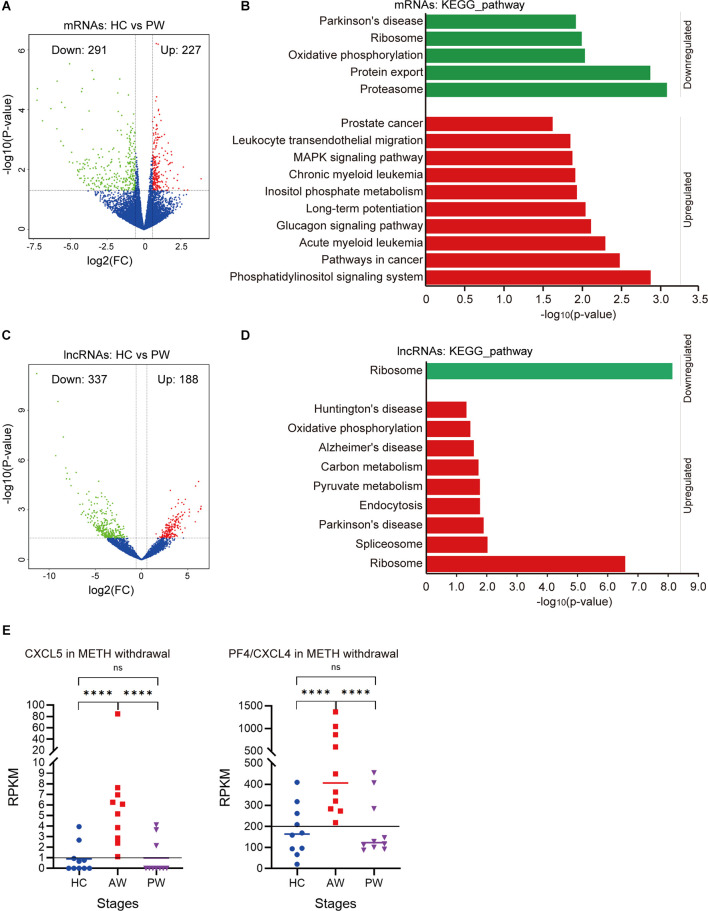
Potential expression profile differences at the AW stage and protracted METH withdrawal (PW) stage. **(A)** Volcano plot showed the DE mRNAs at the PW stage. **(B)** DAVID-KEGG analysis of DE mRNAs at the PW stage. **(C)** Volcano plot showed the DE lncRNAs at PW stage. **(D)** DAVID-KEGG analysis of DE lncRNAs at the PW stage. Green represents downregulated genes; Red represents upregulated genes. lncRNA, long noncoding RNA. **(E)** Potential biomarkers (CXCL5, CXCL4) in the AW stage exosomal transcriptome. ****p < 0.0001; ns, no significant difference.

To determine the health impact of METH dependence after a long-term withdrawal, we further compared the exosomal mRNA/lncRNA profiles between the HC group vs. the PW group. Notably, a total of 518 mRNAs were identified differentially expressed, in which 227 mRNAs were upregulated, and 291 mRNAs were downregulated at the PW stage (Figure 3A). These upregulated mRNAs were significantly enriched in the *Phosphatidylinositol signaling system* (*p* = 0.001), *Pathways in cancer* (*p* = 0.003), *Glucagon signaling pathway* (*p* = 0.007) etc., whereas the downregulated genes were significantly enriched in the *Proteasome* (*p* < 0.001), *Protein export* (*p* = 0.001), *Oxidative phosphorylation* (*p* = 0.009) and *Parkinson’s disease* (*p* = 0.01; Figure 3B and Supplementary Table S3). Compared to the HC group, a total of 188 upregulated and 337 downregulated lncRNAs were identified in the PW group (Figure 3C). Interestingly, the exosomal DE lncRNA at PW also exhibited unique features. DAVID KEGG term analysis suggested that these upregulated lncRNAs were remarkably associated with neurodegenerative diseases, such as *Parkinson’s disease* (*p* = 0.01), *Alzheimer’s disease* (*p* = 0.027), *Huntington’s disease* (*p* = 0.047; Figure 3D and Supplementary Table S3). These results indicated that unlike, METH AW stage, the key difference in exosomal genes/transcripts for patients undergoing protracted METH withdrawal is more likely associated with neurological diseases.

It is noteworthy that the chemokine signaling was remarkably upregulated in the acute METH withdrawal stage (Supplementary Table S2). Further investigation of these DE mRNAs/lncRNAs during the entire withdrawal showed that the expression of CXCL5 and CXCL4 (PF4) was only upregulated in acute METH withdrawal but could be recovered in the protracted withdrawal (Figure 3E). Hence, the expression levels of CXCL5 and CXCL4 in plasma exosomes could be used as potential biomarkers for the METH withdrawal stage.

### Construction of lncRNA-miRNA-mRNA regulatory network associated with METH AW and PW

**Figure 4:**
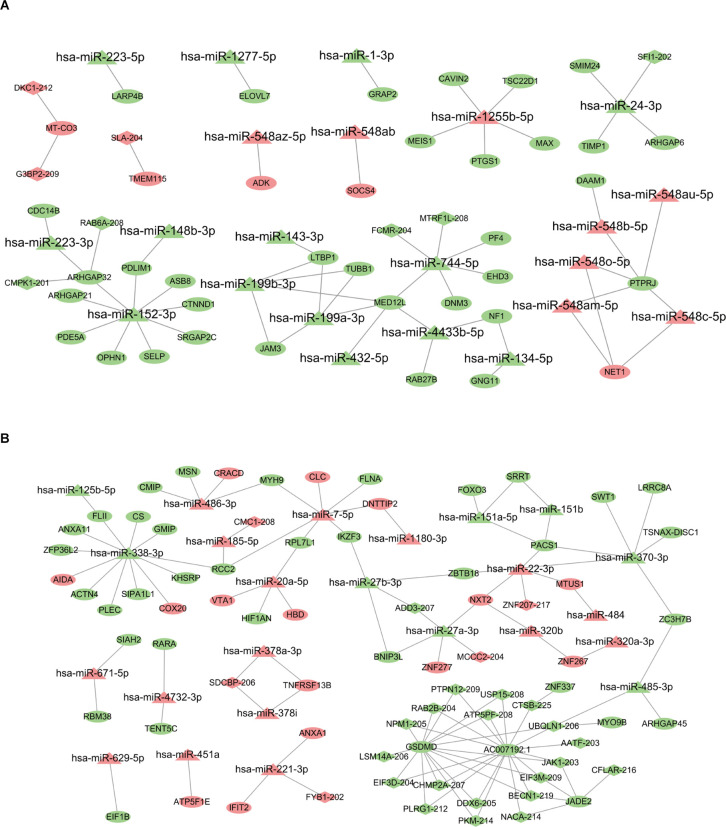
LncRNA-miRNA-mRNA regulatory network associated with AW and PW stage. **(A)** The network related to the AW stage consists of 22 miRNAs, eight lncRNAs, and 38 mRNAs. **(B)** The network related to the PW stage consists of 24 miRNAs, 25 lncRNAs, and 50 mRNAs. Quadrilateral represents lncRNAs, triangle represents miRNAs, and ellipse represents mRNAs.

The DE miRNA has been previously identified in Chen et al. (2021) and Yu et al. (2021). The mRNAs targeted by miRNAs were predicted using miRanda algorithm according to the miRNA-mRNA binding data. For the AW stage, there were 53 miRNA-mRNA target pairs in total were obtained removing duplicates which consisted of 22 miRNAs and 38 mRNAs (Supplementary Table S4). Three significant miRNAs, hsa-miR-152,3p, hsa-miR-1255b-5p, and hsa-miR-744,5p had the most target mRNAs. Next, the lncRNAs regulated by miRNAs were analyzed by miRanda algorithm. Totally, only three miRNA-lncRNA regulatory pairs were identified including three miRNAs and three lncRNAs (Supplementary Table S4). Based on the identified regulatory pairs of miRNA-mRNA and miRNA-lncRNA, a lncRNA-miRNA-mRNA network was constructed, consisting of 22 miRNAs, 8lncRNAs, and 38 mRNAs (Figure 4A). Meanwhile, the lncRNA-miRNA-mRNA network for the METH PW stage was constructed using the same strategy, consisting of 24 miRNAs, 25lncRNAs, and 50 mRNAs (Figure 4B and Supplementary Table S4). Of these miRNA/lncRNA targeted mRNAs, 35 were upregulated, and 15 were downregulated. Four significant miRNAs, hsa-miR-27a-3p, hsa-miR-338,3p, hsa-miR-370,3p, and hsa-miR-7,5p had the most target mRNAs (Supplementary Table S4).

## Discussion

Asa famous psychostimulant drug, METH abuse results in negative consequences, and it is on the rise worldwide (Prakash et al., 2017). Over two decades ago, Jerrells et al. (1989) suggested that withdrawal from alcohol and other substance leads to increased hypothalamic-pituitary-adrenal (HPA) axis reactivity and an acute inflammatory response in the. It is critical because increased acute inflammation may accelerate neurodegeneration and cognitive disability and eventually contribute to high relapse rates and impaired daily functioning. Therefore, to better understand the mechanism associated with developing withdrawal symptoms, it is essential to identify the withdrawal stage-related molecular signatures.

To date, *in vitro* and animal experiments have reported that METH and other substances modulate the function of immune cells, for instance, phagocytosis, chemotaxis, cytokine response, and activity. In particular, METH affects cellular immunity by promoting T cell apoptosis (Iwasa et al., 1996). In the present study, we provide evidence that the CD3+T cells were diminished in the AW stage, indicating that METH dependence interferes with the development of cellular immunity once again (Hernandez-Santini et al., 2021). Harms et al. reported that chronic administration of METH results in a significant decrease in activated T lymphocyte lineage, including both CD4^+^ and CD8^+^T cells (Saito et al., 2006; Harms et al., 2012; Mata et al., 2015; Hernandez-Santini et al., 2021). Our results showed a lower number of CD4^+^T but CD8^+^T cells in the patients undergoing the AW stage. In line with previous findings, these results suggest that decreased number of CD4^+^ helper T cells could diminish the ability to defend against virus infection, such as HIV, making substances more susceptible to HIV infection. However, we didn’t observe a lower number of CD8^+^T cells, probably due to the small number of study participants enrolled. In addition, the immunosuppressive effects of METH were shown in animal models, including decreased NK activity and proliferation rate in splenic lymphocytes (Yu et al., 2002; Akiyama et al., 2008). It has also been reported that METH administration could promote B cell infiltration in the spleen and lung in mice (Mitha et al., 2021). However, we observed no significant change in the number of NK and B cells in patients undergoing METH withdrawal. There is a decreasing trend in B cell numbers in the PW stage. However, these results require further validation in a large group of patient samples.

METH administration in animal models causes microglial activation in key brain regions and contributes to neurodegenerative symptoms (Bowyer and Ali, 2006; Sekine et al., 2008). Notably, IL-1β and TNF-α have been reported to inhibit long-term potentiation by damaging circuit and neuronal plasticity (Cunningham et al., 1996; Butler et al., 2004). Chemokines MCP-1, MIP-1α, and MIP-1β have been shown to play a significant role in neurogenesis and maintain brain function and influence inflammatory processes. Multiple lines of studies in humans have shown neuropsychiatric functions such as mood and cognition may be influenced by cytokines and other immune molecules. Our present data showed that some of the METH acute withdrawal induced cytokine abnormalities such as IL-1β and MIP-1α could be restored to normal at the PW stage. However, the TNFα level of the AW stage was altered and could be only partially restored even a year after the initiation of abstinence. Collectively, these findings support the notion that METH may exert its adverse effects on neuropsychiatric functioning *via* impairing neuroinflammatory regulation.

Recently, exosomes have been identified as a new facet of inter-cellular communications, particularly in neurodegenerative disease studies (Buzas et al., 2014; Thompson et al., 2016). We performed exosomal RNA sequencing and revealed significant changes in mRNA and lncRNA expression profiles at both AW and PW stages, respectively. Interestingly, those specific signaling pathways that are associated with METH withdrawal progression mainly consisted of immune-related chemokine mRNAs and neurological disease-related lncRNAs. Some identified transcripts were previously reported. For example, the genetic variance in UBQLN1 has been recognized as candidate genes for Alzheimer’s disease (Bertram et al., 2005). Another gene ADD3 was discovered by the Psychiatric Genomics Consortium Bipolar Disorder Working Group with a large-scale meta-analysis (Charney et al., 2017). It is worth noting that unlike those reversible changes from the immune system, persistent alterations in the neurological disease-associated lncRNAs were revealed, suggesting that key lncRNAs may play central regulatory roles in mediating severe long-term CNS defects in METH addiction and withdrawal (Aliperti et al., 2021). Our group and others have shown that most immune parameters tested in human opioid abusers are suppressed following withdrawal and recovery time to baseline varies in studies (Eisenstein et al., 2006). The recovery of the immune system after one year of abstinence may be associated with the renewal of immune cells in the body. Regrettably, the hypothesis has not been experimentally determined in patients with MUDs. This novel view and hypothesis updated the previous study of METH and provided a new way to study the effect of METH on the regulatory elements of immunity.

As noted in the literature, numerous experimental models have been applied to explore the knowledge regarding the impartments in the nerve system and the immune system from METH exposure and withdrawal (Loftis and Janowsky, 2014; Potula et al., 2018; Miller et al., 2021). Because the exosomes could cross the blood-brain-barrier (BBB) into the bloodstream, making it possible to extract CNS-derived bioactive substances, the peripheral exosomes have been thought to be promising minimally invasive biomarkers and therapeutic targets for neuronal diseases (Gao et al., 2021; Pineles et al., 2022), multiple lines of evidence indicate that the circulating exosomes might act as the bridge for intercellular communications between neuronal cells (Meng et al., 2020), while contents of these exosomes were closely related to METH addiction status in rats (Li et al., 2018). Notably, screening of all DEmRNAs/lncRNAs during withdrawal found that the CXCL5 and CXCL4 (PF4) were specifically upregulated in the blood exosomes of patients with acute METH withdrawal. Hence, the expression levels of CXCL5 and CXCL4 in blood exosomes may be clinically used as novel biomarkers for the acute METH withdrawal stage. This discovery was in line with the previous finding that CXCL5 was reported remarkably upregulated in the METH-induced astrocyte activation in patients of MUDs at 3 months of withdrawal (Bortell et al., 2017). All the above data suggest that the contents of the peripheral exosomes were clinically valuable biomarkers that could predict or correlate with MUD trajectories and treatment responses to EMTH withdrawal symptoms.

The present study has several strengths. First, the characteristics of recruited patients in the immune cohort and exosome cohorts were systematically analyzed. Second, we divided MUD patients into AW and PW stages of METH withdrawal and identified novel biomarkers. Third, the biomarkers identified were in plasma exosomes rather than in the CNS, so diagnosis was relatively easy. Finally, this study provided in-human evidence that the immune and nervous systems were significantly disrupted in METH AW, whereas the immune systems could e partially restored in the PW stage. The study also has some limitations. The date on dynamic immune and exosomal miRNA/mRNA/lncRNA regulatory networks of MUDs were generated from two independent cohorts, so correlation analysis was not possible. The sample size was relatively small. It is assumed that studies with relatively large sample sizes should obtain more accurate results. The dynamic mRNAs and lncRNAs in peripheral blood exosomes required further studies to determine their origin.

Overall, the present study evaluated the responses of the immune systems and the transcriptomic profiles of the peripheral blood exosomes in patients with MUDs, providing a group of METH withdrawal-related cytokines and exosomal miRNA/mRNA/lncRNA regulatory networks. However additional studies remain needed to determine the further molecular mechanisms underlying the affected immune and nervous systems during METH withdrawal.

## Data Availability Statement

The datasets presented in this study can be found in online repositories. The names of the repository/repositories and accession number(s) can be found below: https://www.ncbi.nlm.nih.gov/, GSE172306.

## Ethics Statement

The studies involving human participants were reviewed and approved by Research Ethics Committee of the First Affiliated Hospital of Kunming Medical University. The patients/participants provided their written informed consent to participate in this study.

## Author Contributions

KW and JY designed the study. ZZ, YM, FC, and GD performed the experiments. FC, PX, and ZX recruited the patients and collected the clinic data. HW, ZZ, and JY analyzed the data, performed statistical analyses, and wrote the manuscript. HW, ZZ, GD, and JY interpreted and discussed the data with all authors. All authors contributed to the article and approved the submitted version.

## Funding

This work was supported by grants from the National Natural Science Foundation of China (Grant No. 81860100, 31860306, 81870458), Science and Technology Department of Yunnan Province (Grant No. 2018DH006, 2018NS0086, 202001AV070010, 2020DAMRA-005, 202002AA100007), Yunnan Province and the Education Department of Yunnan Province (Grant No. 2019J1226), Yunnan Province Clinical Research Center (Grant No. 2019ZF012), Fund of Central Public Welfare Research Institute (Grant No. 2019PT310003), and Yunling Scholar (Grant No. YLXL20170002).

## Footnotes

^1^ http://david.ncifcrf.gov/

^2^ http://www.microrna.gr/LncBase

## Supplementary Material

Supplementary Figure S1 FACS analysis of **(A)** CD3^+^T, **(B)** CD3^+^CD4^+^T, **(C)** CD3^+^CD8^+^T, **(D)** CD16^+^ and CD56^+^NK, and **(E)** CD19^+^B cells in peripheral blood.

Supplementary Table S1 DE mRNAs and lncRNAs between HC and MA groups (AW, PW).

Supplementary Table S2 DAVID_GO and DAVID_KEGG analyses of DE mRNAs and lncRNAs in the AW group.

Supplementary Table S3 DAVID_GO and DAVID_KEGG analyses of DE mRNAs and lncRNAs in the PW group.

Supplementary Table S4 Networks of miRNA-mRNA target pairs at the AW and the PW stage.
