# Supplementary figures and images for: Dynamic immune and exosome transcriptomic responses in patients undergoing psychostimulant methamphetamine withdrawal
Source: Front Cell Neurosci. 2022 Sep 27;16:961131. doi: 10.3389/fncel.2022.961131 (PMC9550894; doi:10.3389/fncel.2022.961131)

Figure S1

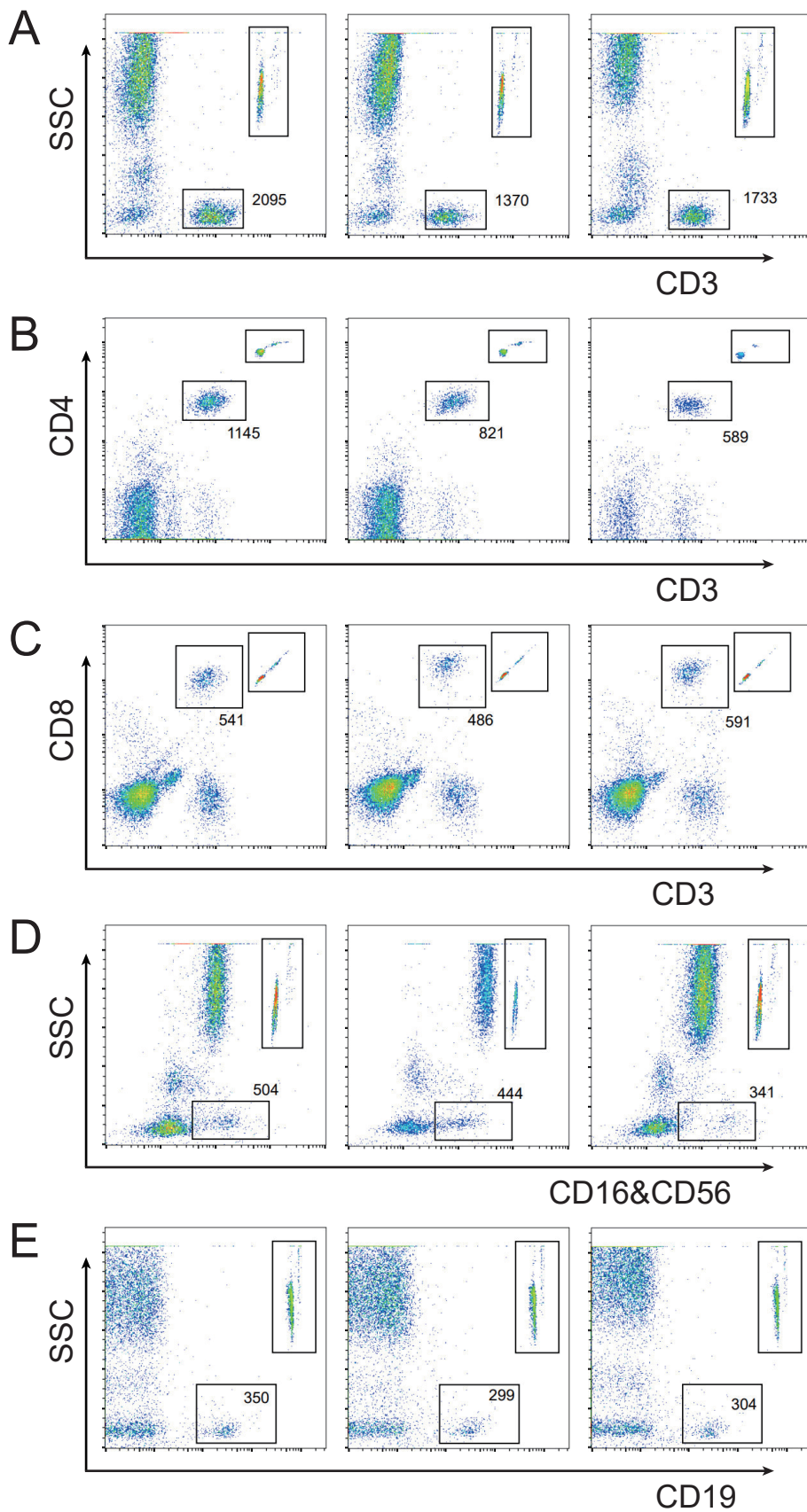

Supplement: Supplementary Figure S1 — FACS analysis of (A) CD3+T, (B) CD3+CD4+T, (C) CD3+CD8+T, (D) CD16+ and CD56+NK, and (E) CD19+B cells in peripheral blood. [file Data_Sheet_1.PDF]
